# Polyaniline-Supported Atomic-Level Pt and Pt-Au Clusters as Catalytic Electrodes in Propanol Oxidation

**DOI:** 10.3390/ma18112594

**Published:** 2025-06-02

**Authors:** Kengo Watanabe, Keisuke Okamoto, Hiroki Kawakami, Shohei Yoshida, Tomoyuki Kurioka, Chun-Yi Chen, Chi-Hua Yu, Yung-Jung Hsu, Takamichi Nakamoto, Masato Sone, Tso-Fu Mark Chang

**Affiliations:** 1 Institute of Integrated Research, Institute of Science Tokyo, Yokohama 226-8501, Japan; watanabe_kengo@ames.pi.titech.ac.jp (K.W.); okamoto@ames.pi.titech.ac.jp (K.O.); kawakami@ames.pi.titech.ac.jp (H.K.); yoshida@ames.pi.titech.ac.jp (S.Y.); kurioka.t.aa@m.titech.ac.jp (T.K.); chen.c.ac@m.titech.ac.jp (C.-Y.C.); yhsu@nycu.edu.tw (Y.-J.H.); nakamoto.t.ab@m.titech.ac.jp (T.N.); sone.m.aa@m.titech.ac.jp (M.S.); 2 Department of Engineering Science, National Cheng Kung University, Tainan 70101, Taiwan; jonnyyu@gs.ncku.edu.tw; 3 Department of Materials Science Engineering, National Yang Ming Chiao Tung University, Hsinchu 30010, Taiwan

**Keywords:** atomic-level metal clusters, Pt, Au, cyclic atomic electrodeposition, catalytic electrode, polyaniline

## Abstract

Noble metals are widely recognized for their ability to catalyze the electro-oxidation of organic compounds, with smaller particle sizes significantly enhancing electrocatalytic activity. In this study, catalytic electrodes decorated with atomic-level platinum and Pt-Au clusters were fabricated using cyclic atomic-metal electrodeposition. The interactions between the iminium (protonated imine) groups in emeraldine salt polyaniline (PANI) and metal chloride complexes in the electrolyte enabled precise control over the cluster size and composition. The electrocatalytic activity of these electrodes for propanol oxidation was systematically evaluated using cyclic voltammetry (CV). Notably, PANI electrodes decorated with odd-numbered atomic-level Pt clusters exhibited higher peak oxidation currents compared to even-numbered clusters, revealing a unique even–odd effect. For atomic-level Pt-Au clusters, the catalytic activity was significantly influenced by the sequence of Pt and Au deposition, with PANI-Au_1_Pt_3_ achieving the highest catalytic activity (35.34 mA/cm^2^). Bi-metallic clusters consistently outperformed mono-metallic clusters, and clusters containing only one Pt atom demonstrated superior catalytic activity. These findings provide valuable insights into the design of high-performance catalytic electrodes by leveraging atomic-level control of the cluster size, composition, and deposition sequence, paving the way for advanced applications in electrochemical sensors.

## 1. Introduction

Electrochemical sensors have emerged as indispensable tools in various fields due to their exceptional advantages, including high sensitivity, excellent selectivity, rapid detection, and low cost. These attributes have facilitated their widespread application in areas such as clinical diagnostics, industrial processes, environmental monitoring, and agricultural analysis [1]. Enhancing the sensitivity of electrochemical sensors remains a critical research focus, with significant efforts dedicated to improving electrode materials. In particular, catalytic electrodes incorporating small-sized noble metal particles—renowned for their high catalytic activity—supported on polyaniline (PANI) have demonstrated significant enhancements in catalytic performance for the electrochemical oxidation of organic compounds, including ethanol, methanol, propanol, and glucose [2,3,4,5,6].

Polyaniline (PANI) stands out as an excellent support material for catalytic clusters due to its high active surface area, decent electrical conductivity, excellent stability, and simple preparation process [7]. The integration of titanium dioxide (TiO_2_) decorated with gold nanoparticles (AuNPs) and PANI as a support material has been shown to ensure the uniform distribution of AuNPs on TiO_2_ and the homogeneous deposition of the resulting AuNP/TiO_2_ composite particles on PANI. This configuration has been demonstrated to further enhance catalytic activity, particularly for glucose oxidation [8]. Palladium has also emerged as a promising catalytic material. For instance, palladium/poly(3,4-ethylenedioxythiophene) (Pd/PEDOT) composite-decorated glassy carbon electrodes (GCEs) have exhibited significantly enhanced catalytic activity for H_2_O_2_ sensing [9].

A key strategy for improving catalytic activity is reducing the size of catalytic materials [2,7,9,10,11]. For example, studies on platinum nanoparticles supported on PANI have revealed that the combination of PANI and platinum nanoparticles exhibits superior catalytic performance [11]. Additionally, comparisons between bulk-sized gold and atomic-level gold have shown that, while bulk-sized gold is catalytically inert in propanol oxidation, atomic-level gold exhibits notable catalytic activity [2]. These findings highlight the potential of reducing the size of Pt clusters supported on PANI to achieve exceptional catalytic properties.

The size effects of noble metals are particularly significant at the atomic scale, where the smallest theoretical size corresponds to the atomic state. Controlling noble metals at the atomic scale offers the potential to develop materials with unique and exceptional catalytic properties for sensor and catalytic applications. A previous study [10] reported the fabrication of atomic-level Au/PANI composite electrodes using a cyclic atomic-metal electrodeposition method. In this method, tetrachloroaurate(III) ions serve as the source of atomic-level Au clusters. The interactions between the iminium groups in emeraldine salt PANI and the gold chloride complexes enable the decoration of atomic-level Au clusters. Specifically, clusters composed of single gold atoms are deposited on the PANI in one cycle, while clusters consisting of two gold atoms are deposited after the second cycle. Interestingly, the catalytic activity of these atomic-level Au clusters for alcohol oxidation revealed a distinct “even–odd effect”, wherein clusters with an even number of gold atoms exhibited higher catalytic activity than those with an odd number [2,5,10]. Similarly, palladium has also demonstrated enhanced catalytic activity when arranged in even-numbered atomic-level metal clusters [12].

Furthermore, the catalytic activity of bi-metallic catalysts differs significantly from that of mono-metallic catalysts [12,13]. Simulation studies have shown that the physical properties of atomic-level bimetallic clusters are highly dependent on their composition [13]. Experimental studies have further demonstrated that combining gold and palladium results in substantial changes in catalytic activity [12]. Notably, even when the ratio of the two metal elements in atomic-level bimetallic clusters is identical, the sequence in which the atoms are deposited has a significant impact on catalytic performance. These findings suggest that mixing different noble metal atoms can yield catalytic activity trends distinct from those observed in mono-metallic systems.

In this study, we investigate the catalytic properties of PANI-supported atomic-level clusters for propanol oxidation. Specifically, PANI-Pt*_N_* clusters (where *N* corresponds to the cycle numbers of the cyclic atomic-metal electrodeposition process conducted and is suggested to represent the atomic size of the cluster, with *N* = 1–6), consisting of atomic-level Pt clusters supported on PANI, and PANI-Pt*_y_*Au*_z_* clusters (where *y* and *z* correspond to the cycle numbers of the atomic electrodeposition process with K_2_PtCl_4_ and KAuCl, respectively, with 1 ≤ *y* + *z* ≤ 4), composed of atomic-level Pt-Au clusters supported on PANI, were fabricated using the cyclic atomic-metal electrodeposition method. The effects of cluster size and deposition sequence on the catalytic activity for propanol oxidation were systematically examined to evaluate their potential as catalytic electrodes in electrochemical sensors.

## 2. Materials and Methods

Aniline (C_6_H_5_NH_2_, 99.5%), perchloric acid (HClO_4_, 70%), tetrafluoroboric acid (HBF_4_, 48%), propanol (C_3_H_8_O, 99.5%), potassium tetrachloroaurate(III) (KAuCl_4_, 99.99%), dipotassium tetrachloroplatinate(II) (K_2_PtCl_4_, 99.99%), and phosphate-buffered saline (PBS, P5493) were purchased from Sigma-Aldrich (Tokyo, Japan). KAuCl_4_ and K_2_PtCl_4_ were used to form the gold chloride complex and platinum chloride complex, respectively. Potassium chloride (KCl, 99.5+%) and potassium hydroxide (KOH, 86+%) were obtained from Kanto Chemical Co. (Tokyo, Japan).

A platinum disk electrode with polyether ether ketone (PEEK) isolation and a diameter of 3 mm (PTE Platinum Electrode, No. 002422, ALS Co., Sagamihara, Japan) served as the working electrode (WE), while a platinum plate with a surface area of 2 cm^2^ was used as the counter electrode (CE). A Ag/AgCl/Sat. KCl electrode (Ag/AgCl, RE-1S, ALS Co., Tokyo, Japan) was employed as the reference electrode (RE).

Prior to PANI deposition, the platinum WE was pretreated in a saturated KCl solution. During the pretreatment, a potential of +1.5 V vs. Ag/AgCl was applied for 180 s while stirring at 400 rpm using a magnetic stirrer. The WE surface was cleaned by rinsing with ultrapure water to ensure complete removal of the residual electrolyte.

PANI was deposited via electropolymerization using an electrolytic solution containing 0.1 M aniline and 2 M HBF_4_, as reported in a previous study [4]. The electropolymerization process consisted of two steps: (1) sweeping the applied potential from 0 to +1.5 V vs. Ag/AgCl at a scan rate of 50 mV/s, followed by (2) applying a constant current of 0.05 mA for 260 s.

**Figure 1:**
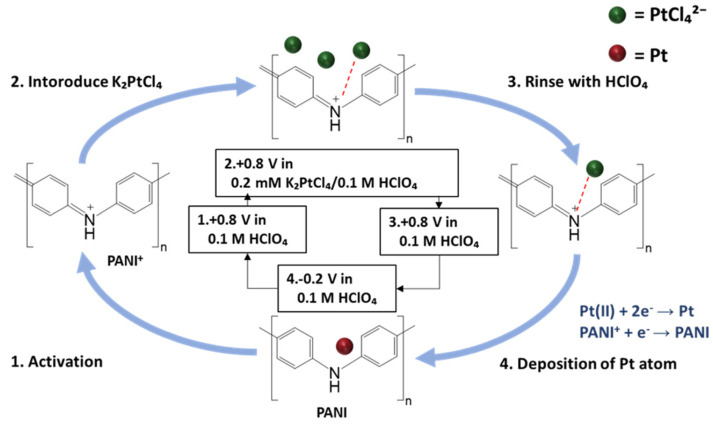
Schematic illustration of one cycle of the atomic-metal electrodeposition process [10,12].

The cyclic atomic-metal electrodeposition process was performed in a three-electrode system, with the PANI-coated Pt electrode as the WE, a Pt foil as the CE, and an Ag/AgCl electrode serving as the RE. Figure 1 illustrates one cycle of the cyclic atomic-metal electrodeposition process, which consisted of the following four steps:

1. Activation step: The base solution was 0.1 M HClO_4_. The base solution was introduced into the flow cell, and the potential was swept from −0.2 V to +0.8 V to activate PANI into its emeraldine salt state.
2. PANI–metal chloride complex formation step: The PtCl_4_^2−^ (or AuCl_4_^−^) was introduced into the flow cell by injecting 0.2 mM K_2_PtCl_4_ (or KAuCl_4_) in the base solution into the flow cell, while maintaining the potential at +0.8 V. During this step, the metal chloride complexes in the base solution were attracted to the iminium groups.
3. Rinsing step: The WE surface was rinsed with the base solution to remove any excess metal chloride complexes that were not bound to the iminium groups, while maintaining the potential at +0.8 V.
4. Reduction step: The potential was swept from +0.8 V to −0.2 V to reduce the metal chloride complexes.

The reduction of one metal chloride complex attached to the iminium group would produce one metal atom. The cycle was repeated to increase the size of the atomic-level metal clusters deposited on the PANI electrode. To fabricate atomic-level bimetallic clusters, the solution introduced into the flow cell was alternated between 0.2 mM KAuCl_4_ and 0.2 mM K_2_PtCl_4_ in the base solution.

**Figure 2:**
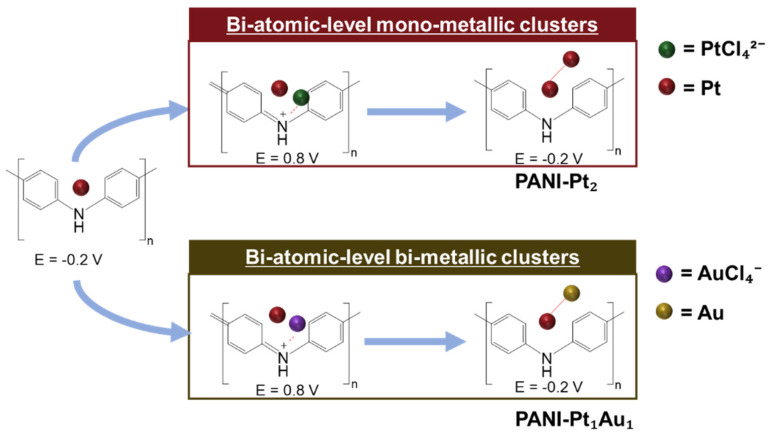
Schematic illustration of bi-atomic-level PANI-Pt_2_ and PANI-Pt_1_Au_1_ clusters decorated on PANI.

Figure 2 schematically illustrates the procedure for decorating atomic-level Pt clusters (e.g., PANI-Pt_2_: Pt deposited in both the first and second cycles) and PANI-Pt_1_Au_1_ clusters (Pt deposited in the first cycle and Au in the second cycle) on the PANI electrode. The naming of the atomic-level metal clusters was based on the sequence of decoration. For example, PANI-Au_1_Pt_1_ refers to bi-atomic-level bi-metallic clusters where Au was deposited in the first cycle and Pt in the second cycle, while PANI-Pt_2_Au_1_ refers to tri-atomic-level bi-metallic clusters where Pt was deposited in the first two cycles and Au in the third cycle.

Cyclic voltammetry (CV) was conducted using a three-electrode system with the atomic-level metal cluster-decorated PANI, a Pt foil, and an Ag/AgCl electrode as the We, CE, and RE, respectively, to evaluate the catalytic activity for propanol oxidation. Prior to the CV, an activation step was performed by subjecting the WE to 10 CV cycles in 1 M KOH within a potential range of −0.6 V to +0.4 V at a scan rate of 100 mV/s. For catalytic activity measurements, an aqueous solution containing 1.3 M propanol and 1 M KOH was used. For the CV, the potential range was from −0.6 V to +0.4 V at a scan rate of 50 mV/s for 20 cycles. The CV curve from the final cycle was used as the result in this study.

## 3. Results and Discussion

**Figure 3:**
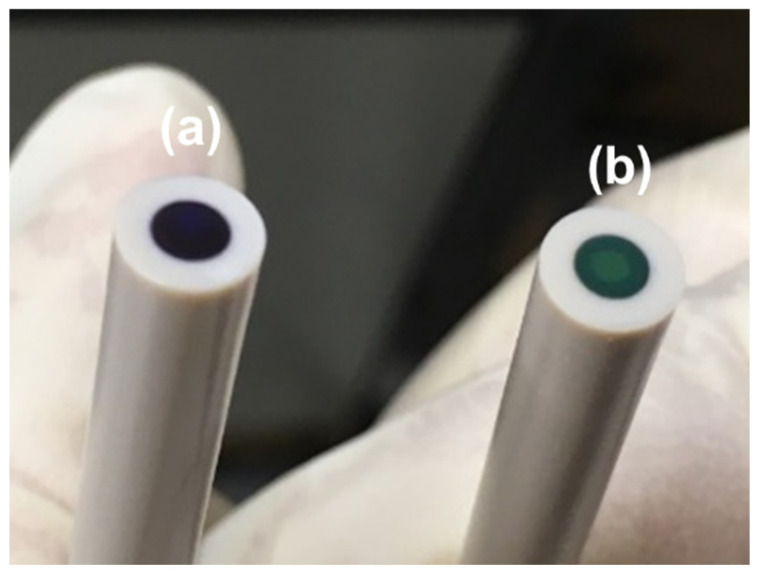
Images of (**a**) as-fabricated PANI on Pt electrode and (**b**) the electrode after the cyclic atomic-metal electrodeposition process.

The as-fabricated PANI on the Pt electrode exhibited a dark color, as shown in Figure 3a. The electrolyte used during the electropolymerization process contained 2 M HBF_4_, and a highly positive potential was applied at the end of the process. Therefore, the dark-colored PANI is likely in the fully oxidized pernigraniline salt form. After undergoing the cyclic atomic-metal electrodeposition process, the color changed to emerald green, as illustrated in Figure 3b. During this process, a 0.1 M HClO_4_ solution was used as the electrolyte, and a potential of −0.2 V was applied at the end of the process. As a result, the PANI transitioned to the emeraldine salt form.

**Figure 4:**
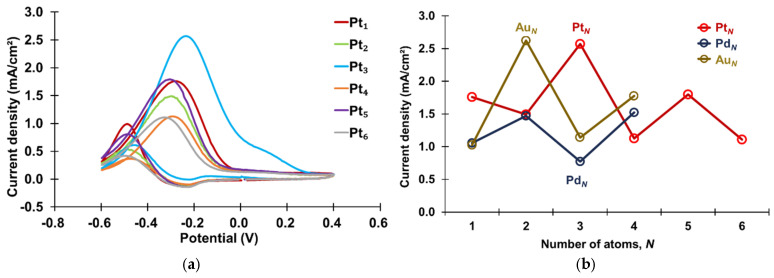
(**a**) CVs of the PANI-Pt*_N_* in an aqueous solution containing 1.3 M propanol and 1 M KOH, recorded at a scan rate of 50 mV/s. (**b**) Peak oxidation current densities of PANI-Pt*_N_*, PANI-Au*_N_* [12], and PANI-Pd*_N_* [12].

The CVs of atomic-level Pt clusters, PANI-Pt*_N_* (*N* = 1–6), in 1.3 M propanol and 1 M KOH solution are presented in Figure 4a. The oxidation current response at approximately −0.3 V during the forward scan corresponds to the oxidation of propanol [14]. Generally, two intermediate reactions are involved in the oxidation of propanol. In the forward scan of the CVs, the first peak corresponds to the oxidation of propanol, with the product suggested to be propionaldehyde. The second peak corresponds to the subsequent oxidation reaction, attributed to the oxidation of propionaldehyde to propionic acid. The peak oxidation current density at approximately −0.3 V was used as an indicator of catalytic activity, with higher values signifying enhanced catalytic performance. The current densities for PANI-Pt_1_, PANI-Pt_3_, and PANI-Pt_5_ were 1.76 mA/cm^2^, 2.57 mA/cm^2^, and 1.80 mA/cm^2^, respectively, whereas those for PANI-Pt_2_, PANI-Pt_4_, and PANI-Pt_6_ were 1.50 mA/cm^2^, 1.13 mA/cm^2^, and 1.11 mA/cm^2^, respectively. These results reveal an even–odd effect in the catalytic activity of atomic-level Pt clusters decorated on PANI, with odd-numbered atomic-level Pt clusters demonstrating higher activity than even-numbered ones. This trend contrasts with the even–odd effect observed for atomic-level Au and Pd clusters in previous studies, where even-numbered mono-metallic clusters exhibited greater catalytic activity [10,12,15]. A comparative analysis of these findings is illustrated in Figure 4b.

The superior catalytic activity of odd-numbered atomic-level Pt clusters is attributed to changes in their electronic structure. Prior studies have shown that the HOMO-LUMO band gap of atomic-level Pt clusters varies quantumly with the number of atoms, with odd-numbered clusters exhibiting a larger band gap and, consequently, higher catalytic activity [16]. Similar trends in HOMO-LUMO band gaps have been reported for Au and Pd atomic clusters, where even-numbered clusters displayed larger band gaps and higher activity [17]. These quantum variations in the HOMO-LUMO band gap likely account for the observed odd–even effect in catalytic activity.

**Figure 5:**
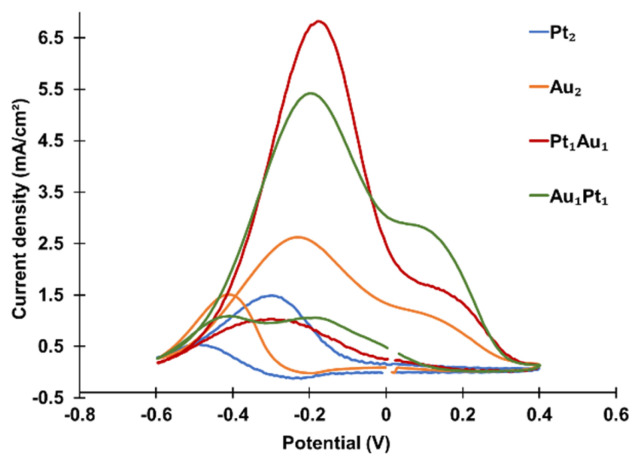
CVs of bi-atomic-level metal clusters decorated on PANI in an aqueous solution containing 1.3 M propanol and 1 M KOH, recorded at a scan rate of 50 mV/s.

Four combinations of bi-atomic-level metal clusters were prepared: PANI-Pt_2_, PANI-Au_2_, PANI-Pt_1_Au_1_ (Pt deposited first), and PANI-Au_1_Pt_1_ (Au deposited first). The CVs of these clusters are shown in Figure 5, and the peak oxidation current densities are summarized in Table 1. Among these, PANI-Pt_1_Au_1_ exhibited the highest catalytic activity, followed by PANI-Au_1_Pt_1_, PANI-Au_2_, and PANI-Pt_2_. The results indicate that bi-atomic-level bi-metallic clusters (PANI-Pt_1_Au_1_ and PANI-Au_1_Pt_1_) exhibit higher catalytic activity than bi-atomic-level mono-metallic clusters (PANI-Pt_2_ and PANI-Au_2_). Regarding the effects of the deposition sequence, PANI-Pt_1_Au_1_ exhibited higher catalytic activity than PANI-Au_1_Pt_1_, suggesting that depositing Pt before Au is beneficial for improving catalytic performance.

**Figure 6:**
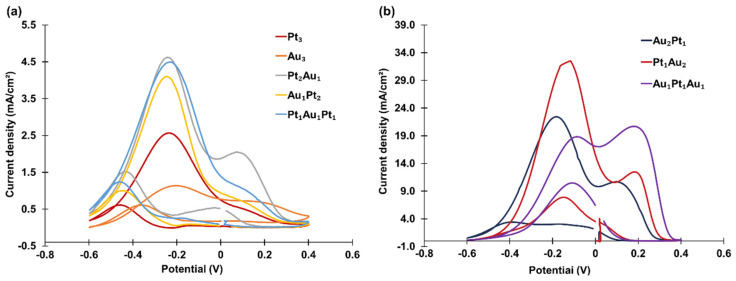
CVs of tri-atomic-level metal clusters with (**a**) zero or more than two Pt atoms and (**b**) one Pt atom decorated on PANI in an aqueous solution containing 1.3 M propanol and 1 M KOH, recorded at a scan rate of 50 mV/s.

Eight tri-atomic-level metal cluster combinations were investigated, including two mono-metallic clusters (PANI-Pt_3_ and PANI-Au_3_) and six bi-metallic clusters: PANI-Pt_1_Au_2_, PANI-Au_1_Pt_1_Au_1_, PANI-Au_2_Pt_1_, PANI-Pt_2_Au_1_, PANI-Pt_1_Au_1_Pt_1_, and PANI-Au_1_Pt_2_. The CVs of these clusters are divided into two groups and presented in Figure 6. In Figure 6a, the tri-atomic-level metal clusters contain zero or more than two Pt atoms, while in Figure 6b, the clusters contain one Pt atom. The corresponding peak oxidation current densities are summarized in Table 2.

Again, the bi-metallic clusters showed higher catalytic activity than the mono-metallic clusters. Comparisons between Figure 6a,b revealed that the catalytic activity was influenced by the composition, with the three tri-atomic-level metal clusters containing one Pt atom exhibiting much higher catalytic activity than the other five tri-atomic-level metal clusters.

**Figure 7:**
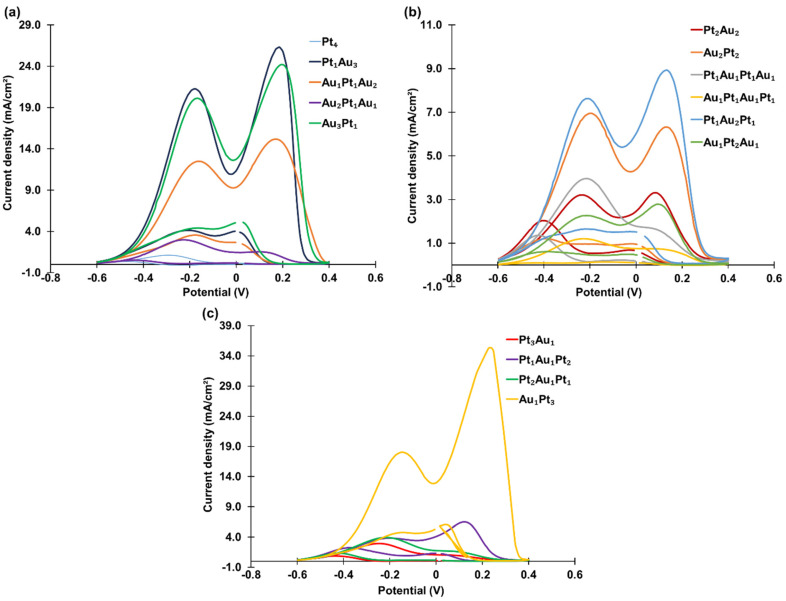
CVs of tetra-atomic-level metal clusters with (**a**) zero Pt atoms and one Pt atom, (**b**) two Pt atoms, and (**c**) three Pt atoms decorated on PANI in an aqueous solution containing 1.3 M propanol and 1 M KOH, recorded at a scan rate of 50 mV/s.

Sixteen tetra-atomic-level metal cluster combinations were studied, including the two mono-metallic clusters (PANI-Pt_4_ and PANI-Au_4_), and bi-metallic clusters such as PANI-Pt_1_Au_3_, PANI-Au_1_Pt_1_Au_2_, PANI-Au_2_Pt_1_Au_1_, and PANI-Au_3_Pt_1_ (one Pt and three Au atoms), as well as PANI-Pt_2_Au_2_, PANI-Au_2_Pt_2_, PANI-Pt_1_Au_2_Pt_1_, and others. The CVs of these clusters are shown in Figure 7, and the current densities are summarized in Table 3.

Among the tetra-atomic-level metal clusters, PANI-Au_1_Pt_3_ exhibited the highest catalytic activity, with a peak current density of 35.34 mA/cm^2^. Bi-metallic clusters consistently demonstrated higher activity than mono-metallic clusters. For instance, PANI-Pt_1_Au_3_ achieved a propanol oxidation current density of 26.32 mA/cm^2^, outperforming both PANI-Pt_4_ and PANI-Au_4_. Among the fourteen tetra-atomic-level bi-metallic clusters, the four clusters composed of only one Pt atom generally showed better catalytic activity, except for PANI-Au_2_Pt_1_Au_1_, which had a current density of only 2.99 mA/cm^2^. The effect of the deposition sequence was not significant as the cluster size increased to four atoms.

## 4. Conclusions

Polyaniline-supported atomic-level Pt and Pt-Au clusters were successfully fabricated as catalytic electrodes for propanol oxidation using cyclic atomic-metal electrodeposition. The precise control over cluster size, composition, and deposition sequence was achieved through the number of electrodeposition cycles and the selection of metal chloride complexes, enabling a systematic evaluation of their catalytic performance.

The study revealed a distinct even–odd effect in the catalytic activity of atomic-level mono-metallic Pt clusters, where odd-numbered clusters (e.g., PANI-Pt_1_, PANI-Pt_3_, PANI-Pt_5_) demonstrated higher catalytic activity compared to even-numbered clusters (e.g., PANI-Pt_2_, PANI-Pt_4_, PANI-Pt_6_). This trend contrasts with the previously reported odd–even effect observed for atomic-level mono-metallic Au and Pd clusters.

For atomic-level bi-metallic Pt-Au clusters, the catalytic activity was significantly enhanced compared to mono-metallic clusters, with the highest current density for propanol oxidation, 35.34 mA/cm^2^, achieved by PANI-Au_1_Pt_3_. The results further showed that bi-metallic clusters containing only one Pt atom generally exhibited superior catalytic activity.

These findings provide valuable insights into the design of high-performance catalytic materials by leveraging atomic-level control of cluster composition, size, and structure. The enhanced catalytic activity of these atomic-level clusters directly translates into improved sensor performance, as higher current densities enable more sensitive detection of target analytes. Furthermore, the ability to fine-tune the composition and deposition sequence of bi-metallic clusters allows for the optimization of sensor response and efficiency. By improving the catalytic efficiency of the electrode materials, this study paves the way for the development of next-generation electrochemical sensors with increased sensitivity, faster response times, and greater reliability, particularly for applications in clinical diagnostics and environmental monitoring systems.

## Figures and Tables

**Table 1:** Summary of the propanol oxidation current densities for bi-atomic-level metal clusters.

Bi-Atomic-Level Metal Cluster		Current Density (mA/cm^2^)
Mono-metallic	Pt_2_	1.50
	Au_2_	2.62
Bi-metallic	Pt_1_Au_1_	7.02
	Au_1_Pt_1_	5.42

**Table 2:** Summary of the propanol oxidation current densities for tri-atomic-level metal clusters.

Tri-Atomic-Level Metal Cluster		Current Density (mA/cm^2^)
Mono-metallic	Pt_3_	2.57
	Au_3_	1.14
Bi-metallic	Pt_2_Au_1_	4.63
	Au_1_Pt_2_	4.11
	Pt_1_Au_1_Pt_1_	4.49
	Au_2_Pt_1_	22.42
	Pt_1_Au_2_	32.51
	Au_1_Pt_1_Au_1_	20.72

**Table 3:** Summary of the propanol oxidation current densities for tetra-atomic-level metal clusters.

Tetra-Atomic-Level Metal Cluster		Current Density (mA/cm^2^)
Mono-Metallic	Pt_4_	1.13
	Au_4_	1.78
Bi-Metallic	Pt_1_Au_3_	26.32
	Au_1_Pt_1_Au_2_	15.18
	Au_2_Pt_1_Au_1_	2.99
	Au_3_Pt_1_	24.20
	Pt_2_Au_2_	3.31
	Au_2_Pt_2_	6.96
	Pt_1_Au_2_Pt_1_	8.93
	Au_1_Pt_2_Au_1_	2.79
	Pt_1_Au_1_Pt_1_Au_1_	1.21
	Au_1_Pt_1_Au_1_Pt_1_	3.96
	Pt_3_Au_1_	2.96
	Pt_1_Au_1_Pt_2_	6.50
	Pt_2_Au_1_Pt_1_	3.96
	Au_1_Pt_3_	35.34

## Data Availability

The raw data supporting the conclusions of this article will be made available by the authors on request.
